# Supplementary material for: Emergence of power and complexity in obstetric teamwork
Source: PLoS One. 2022 Jun 9;17(6):e0269711. doi: 10.1371/journal.pone.0269711 (PMC9182228; doi:10.1371/journal.pone.0269711)
Supplement: S1 Appendix — (DOCX) [file pone.0269711.s001.docx]

# S1 Appendix 1

This is the original interview guide used for the study.

**Interview guide**

**Teamwork as emergent property of obstetric work: a qualitative, exploratory case study**

Principal investigator Dr. med. Christopher Neuhaus

University Hospital Heidelberg

Department of Anesthesiology

Im Neuenheimer Feld 110

69120 Heidelberg

Germany

+49 (0)6221 56 6351

c.neuhaus@uni-heidelberg.de

Principal investigator Dag Erik Lutnæs

Stavanger Acute Medical Foundation for Education and Research (SAFER)

Seehusengate 1

4024 Stavanger

Norway

+47 51 91 10 80

[dagerik.lutnes@safer.net](mailto:dagerik.lutnes@safer.net)

On-site coordinator Dr. Santorino Data

Uganda Department of Pediatrics and Child Health

Mbarara University of Science and Technology

P.O.Box 1410

Mbarara

Uganda

+25 671 221 4458

boymukedata@gmail.com

Supervisor Johan Bergström, Ph.D.

Division of Risk Management and Societal Safety

Lund University

Box 118

221 00 Lund

Sweden

+46 462 220 880

johan.bergstrom@risk.lth.se

#

# Summary

Study title: *Teamwork as emergent property of obstetric work: a qualitative, exploratory case study*

1. **Context, premise and study rationale**

Much of the early safety efforts in medicine was modelled after experiences from the aviation industry, including the implementation of simulation to educate practitioners about human factors. These training programs, so-called ‘crew-resource-management’ (CRM) programs, usually address a number of cognitive and social competencies that are deemed relevant or essential for safety, sometimes dubbed ‘non-technical skills’. Although healthcare is more and more coming to the realization that merely adopting concepts that have proven successful in other domains does little to improve patient safety, there is widespread consensus that teamwork constitutes one of the key requirements in today’s multidisciplinary and highly complex system of delivering care. Many existing frameworks that categorize teamwork apply a normative approach but overlook concepts derived from complexity thinking (e.g. emergence, shared cognition) situated in a joint system. By examining teams that manage peripartum emergencies in different settings and cultures, this study aims to explore how successful teamwork is constructed and perceived by those directly involved in patient care and contrast these findings with traditional normative approaches.

1. **General question**

Is what experienced healthcare providers dealing with peripartum emergencies perceive as good, successful work comparable to normative teamwork approaches communicated through CRM-focused, often simulation based, education?

“What makes up ‘good work’ when dealing with peripartum emergencies? In your opinion, what are the factors that make your work successful?”

1. **General strategy**

In interviews, ask open questions and probe for the informant to tell more about how one perceives

a. collaboration

b. shared mental models

c. coordination

d. communication and

e. leadership

(in accordance with the framework by Manser (2009))

1. **Refinement of the research question**

There are two general issues to study:

1. Explore how successful teamwork is constructed and understood by those directly involved in patient care, and contrast these findings with traditional normative approaches.
2. Considering safety as an emergent property, to highlight cultural differences in the delivery of safe peripartum care between Mbarara (Africa) and Heidelberg (Western Europe)

If possible, we do not want to explicitly ask about teamwork because the term “teamwork” may be biased for the participant. Instead we prefer to ask about how participants perceive ‘good work’ or successful work. What are their thoughts on it, what is important for them?

Manser (2009) pans out the following as “aspects of teamwork relevant to the quality and safety of patient care in dynamical domains of healthcare”. This provides a sufficient base of themes and qualities we would like to probe for.


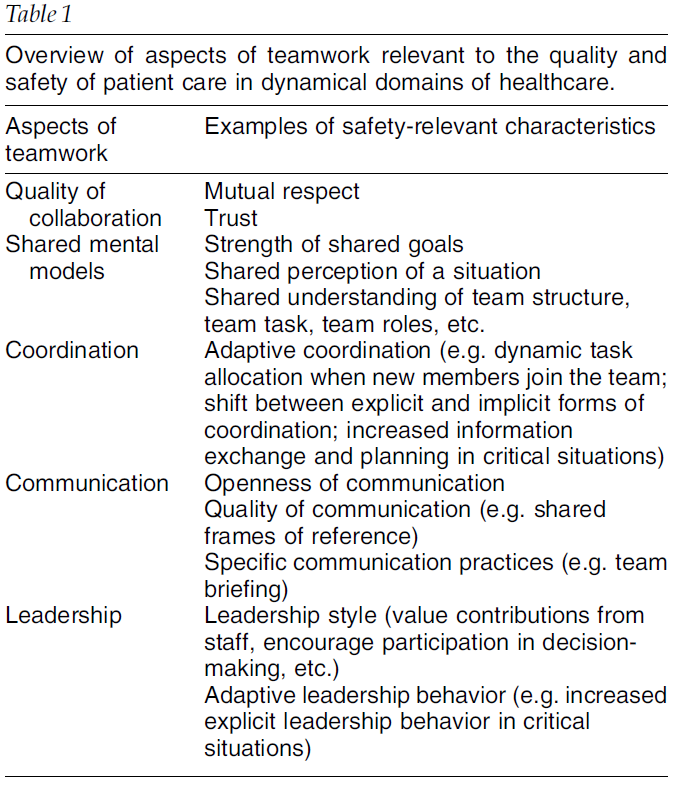


(Manser, 2009, Table 1, p 148)

The aspects found during the interviews related to these themes will be further analysed, processed and compared.

1. **Semi-structured script:**

*“Thank you for agreeing to participate in this study. Our goal is to understand more of how healthcare providers such as yourself perceive good, successful work when dealing with peripartum emergencies (such as postpartum haemorrhage (PPH) or still-births).”*

*“Later on, I may give you some examples of situations that you can respond to. But first,*

*I'd like to learn more about you. My questions are designed to get a sense for how you think*

*about successful work and will provide a context for understanding your responses.”*

1. *“Before we start, could you just remind me:*

- *What is your profession?*
- *What is your speciality (if any)?*
- *What is your current workplace?*
- *Have you always worked there?*
- *How much work-experience do you have?”*

1. *“First, try to think of a time (the last time?) where you experienced a peripartum emergency where the work was successful.*

- *Using your own words, please tell me about it?”*

1. *“What, in your mind, made it successful?”*

Probe for aspects within categories:

- 1. Quality of Collaboration
  2. Shared Mental Models
  3. Coordination
  4. Communication
  5. Leadership

1. *“What makes work successful in general?”*

Probe for aspects within categories:

a. Quality of Collaboration

b. Shared Mental Models

c. Coordination

d. Communication

e. Leadership

1. *“Consider a colleague that you perceive as good and successful in working together with others, which qualities makes you put them in high regard?”*

Probe for aspects within categories:

a. Quality of Collaboration

b. Shared Mental Models

c. Coordination

d. Communication

e. Leadership

1. *“In your opinion, is there a correlation between good work and good outcome for mother and child?”*

# References:

Manser, T. (2009). Teamwork and patient safety in dynamic domains of healthcare: a review of the literature. *Acta Anaesthesiol Scand, 53*(2), 143-151. doi:10.1111/j.1399-6576.2008.01717.x
