# Supplementary material for: Emergence of power and complexity in obstetric teamwork
Source: PLoS One. 2022 Jun 9;17(6):e0269711. doi: 10.1371/journal.pone.0269711 (PMC9182228; doi:10.1371/journal.pone.0269711)
Supplement: S2 Appendix — (DOCX) [file pone.0269711.s002.docx]

S2 Appendix 2: Methodology and audit trail

The purpose of this appendix is to illustrate the methodology used in the study with a higher level of granularity and provide an audit trail for the reader to substantiate our findings. Also, adherence to applicable guidelines from the EQUATOR-network is documented. The results presented in the manuscript were part of a larger research project studying obstetric teamwork in different cultural settings.

[Data collection 2](#_Toc16349181)

[Study design 2](#_Toc16349182)

[Interviews 2](#_Toc16349183)

[Cross-cultural considerations 2](#_Toc16349184)

[Recruitment 3](#_Toc16349185)

[Sample and setting 3](#_Toc16349186)

[Interview questions 4](#_Toc16349187)

[Transcription and translation 4](#_Toc16349188)

[Data analysis 5](#_Toc16349189)

[Methodology for qualitative data analysis 5](#_Toc16349190)

[Analytic strategy and process 5](#_Toc16349191)

[Quantification 7](#_Toc16349192)

[Statistical analysis 7](#_Toc16349193)

[Validity & Reliability 7](#_Toc16349194)

[Biases and limitations 8](#_Toc16349195)

[Ethical considerations 10](#_Toc16349196)

[Declaration of Helsinki 10](#_Toc16349197)

[Ethics review committee 10](#_Toc16349198)

[Data protection and privacy 10](#_Toc16349199)

[Voluntary participation and consent 10](#_Toc16349200)

[Withdrawal of consent 10](#_Toc16349201)

[Reimbursement 11](#_Toc16349202)

[Sponsorship 11](#_Toc16349203)

[Conflicts of interest 11](#_Toc16349204)

[Interviews and demographics 12](#_Toc16349205)

[Consolidated criteria for reporting qualitative studies (COREQ): 32-item checklist 13](#_Toc16349206)

[Domain 1: Research team and reflexivity 13](#_Toc16349207)

[Domain 2: Study design 13](#_Toc16349208)

[Domain 3: Analysis and findings 14](#_Toc16349209)

[References 15](#_Toc16349210)

# Data collection

## Study design

To answer the questions stated in the study objectives, we conducted a qualitative, exploratory case-study [1] at two study sites. Research was conducted at the Heidelberg University Hospital, Heidelberg, Germany, and the Mbarara Medical Simulation Centre/Regional Referral Hospital, Mbarara, Uganda.

#### Heidelberg University Hospital

Heidelberg University Hospital is one of the largest medical centers in Germany, affiliated with the Medical Faculty of the Ruprecht-Karls-University Heidelberg. It serves approximately 2.5 million people in the immediate Rhine-Neckar Metropolitan Region, as well as patients from all across Germany and Europe.

#### Mbarara Regional Referral Hospital

Mbarara Regional Referral Hospital, commonly known as Mbarara Hospital, is a government owned referral hospital in western Uganda. It is affiliated with the Medical School of Mbarara University of Science and Technology (MUST) as primary teaching hospital. The hospital serves a population of over four million people in the area comprising the districts of Mbarara, Bushenyi, Ntungamo, Kiruhura, Ibanda, Buhweju, Rubirizi, Mitooma and Isingiro. The hospital also receives patients from Kabale, Masaka, Fort Portal and neighboring countries like Rwanda and Tanzania.

#### Mbarara Medical Simulation Centre

The Mbarara University of Science and Technology (MUST), the University of Calgary (U of C), the Stavanger Acute Medicine Foundation for Education and Research (SAFER) and the Consortium for Affordable Medical Technologies in Uganda (CAMTech Uganda) have partnered to build a Medical Simulation Centre with the aim of improving maternal, new born and child outcomes in Uganda as part of the ‘Sim for Life’ project.

## Interviews

### Cross-cultural considerations

The researchers are aware of the huge impact of a cross-cultural study design, considering that “[…] local knowledge can be critical to understanding cultural traditions and customs, possible limitations, and the feasibility of the research” [2]. The study protocol was therefore designed in close consideration of recommendations made in the 2016 ‘Guidelines for Best Practice in Cross-Cultural Surveys’ by the University of Michigan Institute for Social Research.

#### Heidelberg

Research in Heidelberg was carried out by Dr. Christopher Neuhaus. Dr. Neuhaus is a German national and has been working as anesthesiologist at the Department of Anaesthesiology, Heidelberg University Hospital, since 2010. He is intimately familiar with local norms and customs.

#### Mbarara

Research in Mbarara was carried out by Dag Erik Lutnæs, a Norwegian national, via videoconference. He was aided by the regional coordinators, Drs. Santorino Data and Lenard Abesiga, both Ugandan nationals. As Course Developer, Project Manager and Senior Facilitator for SAFER, Mr. Lutnæs has been actively involved in the ‘Sim for Life’ project. This included working at Mbarara together with Dr. Data, where he spent weeks training instructors and medical staff at the Medical Simulation Center. Building on this background, he is sensitive to local norms and customs, and was able to apply this knowledge in the interview process.

### Recruitment

Participants were recruited by Dr. Neuhaus in Heidelberg and Dr. Abesiga in Mbarara using convenience sampling as a type of non-probabilistic sampling [3]. Following a three-step procedure for sampling, first the target population was determined by the inclusion criteria specified in the study protocol (see S1 Appendix 1) as follows:

1. Belonging to one of the following professions: Board-certified obstetrician or anesthesiologist, certified or registered nurse or midwife
2. 18 years or older

In a second step, the sample frame was defined by the two predetermined study sites that this project was designed around. Third, individual enrolment was based on subject availability and accessibility, with a target sample size of 10-15 participants per study site. The sample size was in part based on an estimated degree of data saturation, but also based on pragmatic considerations regarding the researchers’ time for as well as the scope of this project. All potential participants were issued the same study information (see S1 Appendix 1) and given time for voluntary consent. To minimize confounding factors, no further selection criteria or restrictions in the number of participants were applied. Nobody who wanted to participate and met the inclusion criteria was excluded.

### Sample and setting

Participants engaged in a semi-structured interview with one of the primary investigators. The interviews were conducted face-to-face in Heidelberg, or via videoconference in Mbarara using a Virtual Meeting Room (Kinly/Skype for Business, Microsoft Inc., Redmond, WA, USA). At the Mbarara site, a private room with videoconference equipment was set up for this purpose at MUST. Interview language was German (Heidelberg) or English (Mbarara). Nobody except the participant and the researcher was present during the interviews.

### Interview questions

To enhance overall data quality, interviews were conducted according to an interview guide that was pre-approved by both Ethics committees (see “Ethical Considerations” below). In addition to general demographic information, participants were asked the following main questions (for a complete description, please see S1 Appendix 1):

- “First, try to think of a time (the last time?) where you experienced a peripartum emergency where the work was successful. Using your own words, please tell me about it?”
- “What, in your mind, made it successful?”
- “What makes work successful in general?”
- “Consider a colleague that you perceive as good and successful in working together with others, which qualities makes you put them in high regard?”
- “In your opinion, is there a correlation between good work and good outcome for mother and child?”

### Transcription and translation

The interviews were audio-recorded, and recordings were later transcribed using f4transcript for Mac® (dr. dresing & pehl GmbH, Marburg, Germany) and Express Scribe® (NCH Software Inc., Greenwood Village, CO, USA). Interviews from Heidelberg were translated into English by the primary site investigator, who has lived, studied and worked in the United States of America and is fluent in both general and medical English. All personal or identifying information was removed during the transcription process.

# Data analysis

## Methodology for qualitative data analysis

As our study is only partly confirmatory and mostly exploratory in nature, data was analyzed using Applied Thematic Analysis (ATA). The ATA approach is “[…] a rigorous, yet inductive, set of procedures designed to identify and examine themes from textual data in a way that is transparent and credible” [4]. Drawing from a multitude of theoretical and methodological perspectives, its “[…] primary concern is with presenting the stories and experiences voiced by study participants as accurately and comprehensively as possible” [4].

## Analytic strategy and process

Guest, MacQueen [4] describe data analysis in applied thematic analysis as “locating meaning in the data”. Distinct to the often-encountered idea of sensemaking in qualitative research, this reinforces a measured approach that is cautious of highly imaginative over-interpretation of problematic data. Our analytic strategy was therefore designed according to recommendations by Guest, MacQueen [4] with the main goal of providing an “audit trail” of the process rather than an analytic “black box” that leaves many questions up to the reader’s imagination. More precisely, this audit trail starts at the description of how we arrived and chose a research question, follows all through the methods section and ends with our analysis and conclusions. It is supported by the appendices, which we invite the reader to explore to answer any remaining questions. While we hope to illustrate the process for the sake of validity and transparency, there will always be complex workings of the researcher’s mind at play that defy simple description. Especially in the later stages of coding, when faint traces of meaning are located and analytic choices are made, codes, definitions and analytic barriers sometimes need to be revisited and changed in light of new findings. We rely on our audit trail to tell the story. All qualitative data analysis, as well as data visualization, note taking, recording of memos and analysis ideas was performed using NVIVO 12 for Mac ® (QSR International, Melbourne, Australia).

In our study, the iterative process of data analysis consisted of the following phases:

1. *Establishing clear analytic objectives*: The main purpose of our study was to harness the narratives of practitioners and develop an understanding for their conceptualization of teamwork. This is highly exploratory in nature, and due to logistical and organizational constraints limited to a comparative case-study of two independent sites.
2. *Data quality control and enhancement during data collection*: During the interview process, the two researchers frequently exchanged experiences and compared notes regarding the interviews. This served two important functions: First, to control the quality of data gathered and ensure the reliability between the two interviewers, and second to already familiarize oneself with the data and develop a ‘feel’ for recurring themes and ideas voiced by the participants.
3. *Text segmentation and quality control:* After transcription, interview data was segmented by questions as specified in the interview guide. This was later used to assess and compare the consistency of the questions asked and to provide a foundation for comparability. This segmentation was purely structural in nature and meant to support our methodological approach. We were especially conscious of the ongoing controversy regarding text segmentation and its potential for distorting context and meaning [5], therefore the original interview dialogue was preserved and used for all coding and analysis of content.
4. *Development of initial themes and codes*: As the stated primary objective of our study is to “explore how successful teamwork is constructed and understood by those directly involved in patient care and contrast these findings with traditional normative approaches”, we applied different strategies in the initial mapping of our data: Both researchers together looked for representations of teamwork aspects as described by Manser [6, see Table 2] in the participant’s narratives, while at the same time generating emergent themes and defining vague boundaries around them.
5. *Development of a codebook and content coding*: In the next iteration, themes were more closely defined, restructured, and a codebook developed for definite coding of the text (see S3 Appendix 3). Subsequently, all interviews were coded for content by both researchers together. This process was repeated twice, where the codebook was further refined, and analysis notes, ideas and memos were recorded for later analysis. It is important to note at this point that steps 3 – 5, while easily broken down into a logical sequence of events, can hardly represent distinct processes. Guest, MacQueen [4] note that “[…] the act of identifying a meaningful segment of text calls for some minimal representation of that meaning as a code, a note, a query, or a tag”. Subsequently, coding ideas come to mind even during the initial reading and structural segmentation of the text. On the other hand, codes that were conceived and hardly ever applied were later changed, or redefined, as the process (r)evolved.
6. *First-order data analysis and comparison:* Subsequently, we analyzed data by location to provide a description of how teamwork was constructed by our participants. Moreover, comparisons between Heidelberg and Mbarara were made by extracting different codes and looking at the content in greater detail. Also, quantification methods were used, mostly to visualize code frequencies and aid the researchers in pattern recognition.
7. *Second-order analysis:* During the second-order analysis, the sometimes incomplete, sketchy or contradictory data is connected with theoretical literature with the aim of constructing theoretical explanations. According to Shkedi [7], the purpose of these explanations is not necessarily the construction of “grand theory”, but rather a conversion and organization of the content and the connection with the researcher, as it is impossible to separate the inquirer from the inquired from a constructivist epistemological perspective. For us, this not only meant making sense of the analysis, but aligning the results with our own experience and understanding of the delivery of peripartum care.
8. *Writing the report:* Rather than a mere form of representation, writing “[…] plays an active part in the process of organizing, working with and analyzing data” [5]. It is also a way of crystallizing vague conceptions and connecting loosely formulated ideas, and presenting them to the reader through sound reasoning and exemplification. But above all, the cardinal rule of writing according to Guest, MacQueen [4] is to avoid annoying your audience without becoming polemic. You be the judge.

### Quantification

As previously noted, some quantification of qualitative data was performed in order to aid in pattern recognition and visualization of data. This type of mixed-method approach is sometimes questioned, if not frowned upon, in classical qualitative research, as it is seen as dichotomous to qualitative approaches. We exercised extreme caution so as not to create new data without meaning, or infer any statistical calculations or significances. However, quantifiable measures like code frequencies or word counts can be understood as supplemental analytic techniques that enhance a thematic analysis [4]

### Statistical analysis

Quantitative data analysis is restricted to descriptive analysis of the participants (occupation, experience) and the interview process (number of interviews, average length). Analysis was performed using Microsoft Excel for Mac 2011 ® (Microsoft, Redmond, WA, USA).

### Validity & Reliability

A myriad of definitions try to capture the essence of validity in research, always revolving around issues of accuracy of measurement, approximation of reality and trustworthiness of the results. “An account is valid or true if it represents accurately those features of the phenomena that it is intended to describe, explain or theorize” [Hammersley, 1987, p.69, as cited by 4]. Reliability, also oftentimes referred to as consistency or dependability, on the other hand, is a conditio sine qua non for validity. “Since there can be no validity without reliability (and thus no credibility without dependability), a demonstration of the former is sufficient to establish the latter.” [8]. The more pressing question becomes how validity can be demonstrated for a particular type of research. The usual solution in quantitative inquiry is the comparison of one’s own variables to pre-established standards while assuming their “credibility”, thereby creating an inescapable tautology. In qualitative inquiry, validity is much more reliant on research and analysis procedures, reinforcing the need for transparency to “[…] making a convincing case for the validity of one’s findings and interpretations” [Miles & Huberman, 1994, p. 278, as cited by 4].

In addition to the aforementioned audit trail, we took the following steps to increase the validity of our research during various stages of the project:

- The study design, including the format and questions of the semi-structured interview, was developed by both principal researchers together. This ensured familiarity with the research objectives and the envisioned methods for data acquisition.
- The interviews were conducted by the same researchers that designed the study, therefore the purpose of each question was known as prerequisite for further inductive probing. During the interview phase, we constantly monitored and compared data as it was collected, so probing techniques could be aligned and overall data consistency enhanced.
- Transcriptions were made to provide verbatim accounts of the data collection event, including the captioning of reactions (thought phases, pauses, laughs, sighs, etc.). In addition to providing more precise accounts of the interviews than mere field notes, we were able to capture and document rich, powerful and sometimes very emotional quotes that convey much more meaning than any analysis ever will. In light of our multi-site research, transcripts form the necessary basis for reliable data comparison.
- A codebook was developed through multiple iterations. Also, coding was conducted together, so that ambiguities and reliability problems due to different interpretations could be immediately resolved.
- Where possible, we tried to employ triangulation techniques by using supplemental data sources to build our argument.

### Biases and limitations

Our study, of course, has several limitations. For starters, the overall scope of the project, and the decision to pick two study sites, establish clear boundaries in terms of generalizability of our findings. We were always aware that it is an exploratory case-study that might prove to be hypothesis-generating at best, and descriptive of local, insulated and circumstantial phenomena at least. However, through the use of additional data sources and triangulation, we try to provide some frame of reference for the reader to put our findings into perspective. This is also the reason why we try to harness another source of potential bias, our own domain knowledge in healthcare and medical simulation, and for one of us (CN), our continuing involvement in the active management of peripartum emergencies as part of our professional duties. As stated in the introduction, we are very much aware of this lack of independence; however, our underlying motivation should go a long way of reinforcing our efforts at dealing with this bias and its potential influence on the results.

Interviewing study participants, no matter how well scripted, will elicit biased responses, based on a multitude of factors. Each study site brought with it its own peculiarities:

- In Mbarara, the use of videoconferencing, paired with sometimes subpar audio quality and language issues, might have influenced results. Also, the investigator had visited and worked there on two occasions, and was therefore vaguely acquainted with some of the participants.
- In Heidelberg, all study participants were at least vaguely acquainted with the investigator, and some had previously worked with him on several occasions.

When reading through the interviews, however, the honest, critical and open responses to our questions can be seen as testament to the participant’s professionalism, and to the atmosphere and setting the interviews were conducted in. We like to believe that our questions “struck a nerve”, and gave participants a chance to tell stories they felt worth telling.

In this regard, it was helpful for the process of reflexivity that in both cases, the other researcher was impartial, and the joint analysis and coding could help reveal and deal with biased interpretation. This is also one reason why we chose to do all the coding and analysis work together, thereby accepting the fact that we would not be able to calculate a Cohen’s Kappa as formal inter-rater reliability score, but had to revert to a mere subjective assessment. Also, our analysis work was not subject to external review.

## Ethical considerations

###

### Declaration of Helsinki

The study was conducted in accordance with the Declaration of Helsinki. The declaration delineates ethical principles for medical research involving human subjects, among those dignity, integrity, right to self-determination, privacy, and confidentiality of personal information of research subjects. It also demands proper scientific conduct, the use of protocols, and review and approval by independent ethics review committees. In regard to this thesis, these aspects will be addressed in the following paragraphs.

###

### Ethics review committee

Before commencing the study, approval of the study protocol (see S1 Appendix 1) has been granted from both the Ethics Review Committee of the Medical Faculty of the Ruprecht-Karls-University Heidelberg (Ref: S-110/2018) and the Mbarara University of Science and Technology – Research Ethics Committee (Ref: MUREC 1/7).

### Data protection and privacy

Numerical codes and pseudonyms were assigned to each participant to maintain confidentiality. All identifying information were removed from the transcripts. Information linking participants to pseudonyms are kept in a locked file on encrypted USB drives that can only be accessed by the two principal investigators. Interview recordings were destroyed at the completion of the study. Consent forms were stored on encrypted USB drives (electronic) or in proprietary folders (paper) and kept in locked file cabinets in the offices of both primary investigators. Data collection was conducted in private settings as described above. The names of the informants and all other confidential information is subject to medical confidentiality. In Germany, federal and state laws and regulations regarding the use of such data apply (Landesdatenschutzgesetz Baden-Württemberg LDSG BW und Bundesdatenschutzgesetz BDSG).

### Voluntary participation and consent

Participants’ involvement in this study, as well as the permission to collect, use and share data was strictly voluntary. Participants were thoroughly educated both verbally and with written information about the planned study and its effects, with special emphasis on the risks associated with participation. This took place before scheduling the interview. Written consent was obtained from participants prior to their involvement in the study.

### Withdrawal of consent

Participants could decide to withdraw their consent at any given time. At the request of the individual, all existing interview recordings, transcripts and written notes would be exempt from the analysis and destroyed. None of the participants chose to withdraw consent.

### Reimbursement

Participants were not reimbursed and were offered no incentives for participation in this study. In Mbarara, in keeping with local customs, a small token of appreciation of an educational nature with a value not exceeding 5 USD was offered to the participants.

### Sponsorship

The primary investigators did not receive any funding for the research proposed in this study protocol.

### Conflicts of interest

This project work is in partial fulfilment of the requirements for the MSc in Human Factors and Systems Safety at Lund University for both principal investigators, who are students in the program.

## Interviews and demographics

Interviews were conducted between June and November 2018. A total of 13 healthcare professionals in Heidelberg and 14 in Mbarara chose to participate in our study. Table 1 provides an overview of participants’ roles and experience.

**Table 1**: Participants’ roles and experience

|  |  | |  | Heidelberg | Mbarara |
| --- | --- | --- | --- | --- | --- |
| No. of participants | |  | | 13 | 14 |
| Roles | | Obstetrician | | 5 | 3 |
|  |  | Midwife | | 6 | 10 |
|  |  | Anesthesiologist | | 2 | 0 |
|  | | Pediatrician (w/ OB experience) | | 0 | 1 |
| Experience | | 0 - 2 years | | 1 | 0 |
|  |  | 3 - 5 years | | 3 | 6 |
|  |  | 6 - 10 years | | 7 | 5 |
|  |  | 11 – 20 years | | 2 | 3 |

In Heidelberg, 8 individual interviews and two group interviews were conducted, as 2 and 3 midwives, respectively, preferred to be interviewed as a group. In Mbarara, 14 individual interviews were conducted. Mean interview duration was 30.4 minutes (Heidelberg 29.6 minutes, Mbarara 31.0 minutes, SD 6.8 minutes). Regarding adherence to the interview guide, during two interviews in Mbarara the last question could not be asked/answered due to connectivity issues. All other 25 interviews followed the interview script. As the interview questions #1-3 built on one another, on three occasions participants already intuitively gave answers to subsequent questions during the conversation, which led the interviewer to not explicitly ask these questions again. This happened twice in Heidelberg with question #2 and once in Mbarara with question #3. All instances were reviewed by the research team, which concluded that the interview content followed the logic of the pre-structured script and that structural reliability was not compromised. Overall, 93% of the interviews showed the structure specified in the interview guide. No repeat interviews were carried out.

# Consolidated criteria for reporting qualitative studies (COREQ): 32-item checklist

The COREQ checklist [9] provides a consolidated reporting framework for qualitative research. All items were addressed above or in the manuscript; exceptions are commented on below.

### Domain 1: Research team and reflexivity

*Personal Characteristics*

1. Interviewer/facilitator: Which author/s conducted the interview or focus group? *See S2 Appendix 2/Interviews/Heidelberg (p. 3)*
2. Credentials: What were the researcher’s credentials? *See above*
3. Occupation: What was their occupation at the time of the study? *See above*
4. Gender: Was the researcher male or female? *Male, see above*
5. Experience and training: What experience or training did the researcher have? *See above*

*Relationship with participants*

1. Relationship established: Was a relationship established prior to study commencement? *Manuscript/Limitations (p.19):* *All study participants were at least vaguely acquainted with the investigator, and some had previously worked with him on several occasions.*
2. Participant knowledge of the interviewer: What did the participants know about the researcher? *See above*
3. Interviewer characteristics: What characteristics were reported about the interviewer/facilitator? *See above*

### Domain 2: Study design

*Theoretical framework*

1. Methodological orientation and theory: What methodological orientation was stated to underpin the study? *See Manuscript/Methods (p. 5) and S2 Appendix 2/Data Analysis/Methodology (p. 5)*

*Participant selection*

1. Sampling: How were participants selected? *see S2 Appendix 2/Data Collection/Recruitment (p. 3)*
2. Method of approach: How were participants approached? *see S2 Appendix 2/Data Collection/Recruitment (p. 3)*
3. Sample size: How many participants were in the study? *see S2 Appendix 2/Data Collection/Recruitment (p. 3, 2^nd^ paragraph)*
4. Non-participation: How many people refused to participate or dropped out? Reasons? *None, see S2 Appendix 2/Data Collection/Recruitment (p. 3)*

*Setting*

1. Setting of data collection: Where was the data collected? *see S2 Appendix 2/Data Collection/Sample and Setting (p. 3)*
2. Presence of non-participants: Was anyone else present besides the participants and researchers? *See above*
3. Description of sample: What are the important characteristics of the sample? *See Manuscript/Participants and demographics (p. 6)*

*Data collection*

1. Interview guide: Were questions, prompts, guides provided by the authors? Was it pilot tested? *see S2 Appendix 2/Data Collection/Interview questions (p. 3)*
2. Repeat interviews: Were repeat interviews carried out? If yes, how many? *No, see* *Manuscript/Participants and demographics (p. 6)*
3. Audio/visual recording: Did the research use audio or visual recording to collect the data? *see S2 Appendix 2/Data Collection/Transcription and translation (p. 4)*
4. Field notes: Were field notes made during and/or after the interview or focus group? *No, only audio recordings, see S2 Appendix 2/Data Collection/Transcription and translation (p. 4)*
5. Duration: What was the duration of the interviews or focus group? *No, see* *Manuscript/Participants and demographics (p. 7)*
6. Data saturation: Was data saturation discussed? *Yes, see* *S2 Appendix 2/Data Collection/Recruitment (p. 3)*
7. Transcripts returned: Were transcripts returned to participants for comment and/or correction? *Transcripts were not returned to participants for comment or correction.*

### Domain 3: Analysis and findings

*Data analysis*

1. Number of data coders: How many data coders coded the data? *Two, see* *S2 Appendix 2/Data Analysis/Methodology (p. 6, items 3-7)*
2. Description of the coding tree: Did authors provide a description of the coding tree? *See S3 Appendix 3*
3. Derivation of themes: Were themes identified in advance or derived from the data? *Both, see S2 Appendix 2/Data Analysis/Methodology (p. 5-6)*
4. Software: What software, if applicable, was used to manage the data? *NVIVO 12 for Mac, see* *Manuscript/Methods (p. 6)*
5. Participant checking: Did participants provide feedback on the findings? *Several informants inquired about the findings and generally agreed with the conclusions.*

*Reporting*

1. Quotations presented: Were participant quotations presented to illustrate the themes / findings? Was each quotation identified? *Yes, see explanation under Manuscript/Methods (p. 7, 1st paragraph)*
2. Data and findings consistent: Was there consistency between the data presented and the findings? *Yes, see Manuscript/Results (p. 7ff)*
3. Clarity of major themes: Were major themes clearly presented in the findings? *Yes, see Manuscript/Results (p. 9-12ff)*
4. Clarity of minor themes: Is there a description of diverse cases or discussion of minor themes? *Yes, see Manuscript/Results (p. 9-12ff)*

# References

1. Yin, R.K., *Qualitative Research from Start to Finish, Second Edition*. 2015, New York, NY: Guilford Publications.

2. Survey Research Center, *Guidelines for Best Practice in Cross-Cultural Surveys*, University of Michigan Institute for Social Research, Editor. 2016: Ann Arbor, MI.

3. Elliot, M., et al. *non-probability sampling*. 2016; Available from: <http://www.oxfordreference.com/view/10.1093/acref/9780191816826.001.0001/acref-9780191816826-e-0280>.

4. Guest, G., K. MacQueen, and E. Namey, *Applied Thematic Analysis*. 2012: Thousand Oaks, California.

5. Gibson, W.J. and A. Brown, *Working with Qualitative Data*. 2009: London.

6. Manser, T., *Teamwork and patient safety in dynamic domains of healthcare: a review of the literature.* Acta Anaesthesiol Scand, 2009. **53**(2): p. 143-51.

7. Shkedi, A., *Second‐order theoretical analysis: a method for constructing theoretical explanation.* International Journal of Qualitative Studies in Education, 2004. **17**(5): p. 627-646.

8. Guba, E.G. and Y.S. Lincoln, *Naturalistic inquiry*. 1985, Sage: Newbury Park, CA.

9. Tong, A., P. Sainsbury, and J. Craig, *Consolidated criteria for reporting qualitative research (COREQ): a 32-item checklist for interviews and focus groups.* International Journal for Quality in Health Care, 2007. **19**(6): p. 349-357.
