# Supplementary material for: Emergence of power and complexity in obstetric teamwork
Source: PLoS One. 2022 Jun 9;17(6):e0269711. doi: 10.1371/journal.pone.0269711 (PMC9182228; doi:10.1371/journal.pone.0269711)
Supplement: S3 Appendix — (DOCX) [file pone.0269711.s003.docx]

# S3 Appendix 3: Codebook

| Name | Description |
| --- | --- |
| Themes | General themes recognized in the interviews |
| Emergent |  |
| Experience | Overall work experience in the medical context. If only related to medical expertise, see medical competency |
| Perception | How work is perceived |
| Personality | Personality traits or attributes considered important for good teamwork |
| Approachability | generally referred to as „easy to talk to“ |
| Attitude | attitude towards work, work ethic |
| Medical competence | Knowledge, skills, etc… |
| Reflexivity | Introspection, being critical of one’s own actions |
| Self-reliance | Reference to one’s own ability of coping with situations |
| Systems view | things like no-blame, new view attitudes as expressed by the interviewee |
| Power |  |
| 1st dimension | power as strategically exercised through intentional, rational calculation, e.g. position power, information, expertise, coercion |
| 2nd dimension | more covert form of conflict, non-decisions, … |
| 3rd dimension | hinges on the construction of meaning in social life, and enables the dominant to influence the dominated to adopt the goals, values and attitudes of the dominant. |
| Hierarchy |  |
| formal vs. informal | how hierarchy is enacted in real-life situations, usually as opposed to formal definitions |
| Positive h. | Hierarchical aspects of work that are positively perceived by the interviewee |
| Problematic h. | Hierarchical aspects of work that are negatively perceived/perceived as problematic by the interviewee |
| Role transfer | Shifting relationships between formal roles and hierarchical positions |
| Resources | Reference to available resources (infrastructure, people, equipment, …) |
| Standardization | Using Guidelines, SOP, etc.. |
| WAI WAD | Work descriptions where differences from WAI are expressed |
| Teamwork | Mentions/explanations of teamwork in the interviewee’s own words |
| Manser (2009) | aspects of teamwork relevant to the quality and safety of patient care in dynamical domains of healthcare as defined by Manser (2009) |
| Communication | Openness of communication, Quality of communication (e.g. shared frames of reference), Specific communication practices (e.g. team briefing) |
| Coordination | Adaptive coordination (e.g. dynamic task allocation when new members join the team; shift between explicit and implicit forms of coordination; increased information exchange and planning in critical situations) |
| Preparedness | also planning ahead |
| Leadership | Leadership style (value contributions from staff, encourage participation in decision- making, etc.), Adaptive leadership behavior (e.g. increased explicit leadership behavior in critical situations) |
| Quality of collaboration |  |
| Appreciation | a positive form of respect, can be interpersonal or professional |
| Flexibility | Openness, flexibility to change way of working, procedures etc. |
| Interpersonal relationship | Interpersonal as opposed to professional->see collaboration |
| Mutual respect | can be neutral/professional, respect for someone’s qualifications, work, contribution etc. |
| Mutual support | Helping others by doing their jobs, supporting, etc. |
| Reliability | „true to one’s word“ |
| Trust |  |
| Shared mental models | Strength of shared goals, Shared perception of a situation, Shared understanding of team structure, team task, team roles, etc. |
| System | systemic issues, like quality vs. safety etc. |
