# Supplementary material for: Emergence of power and complexity in obstetric teamwork
Source: PLoS One. 2022 Jun 9;17(6):e0269711. doi: 10.1371/journal.pone.0269711 (PMC9182228; doi:10.1371/journal.pone.0269711)
Supplement: Attachment — Submitted filename: Evaluation form (1).doc [file pone.0269711.s004.doc]

**Manuscript Evaluation Form**

| **PAPER TITLE** |  |
| --- | --- |
| **AUTHOR(S) NAME** |  |

***Sec. A: REFEREE'S ASSESSMENT***	(cross as appropriate)

| **Criterion** | **Excellent** | | **Good** | | | **Fair** | **Poor** |  |
| --- | --- | --- | --- | --- | --- | --- | --- | --- |
| Originality of the Topic |  | | X | | |  |  |  |
| Technical Quality |  | | X | | |  |  |  |
| Importance in its Field |  | | X | | |  |  |  |
| Style & Overall Representation |  | | X | | |  |  |  |
| Readily Understandable |  | | X | | |  |  |  |
| Suitability for the Journal |  | |  | | | X |  |  |
| Adequate Illustrations or Drawings |  | |  | | | X |  |  |
| English Language |  | | X | | |  |  |  |
| **Description** | | **Yes** | | **No** | **Comments/Suggestions** | | | |
| Does the title represent manuscript's contents? | | **X** | |  |  | | | |
| Is the Abstract accurate and concise? | |  | | **X** | **It contains some inconsistent findings. For, e.g. They stated, “**Discontinuation of implanon was associated with lack of counselling during service delivery 2.54 times (OR: 2.34, 95%CI: 2.98, 2.77)**, which is not valid.** | | | |
| Are the approaches/methods properly described? | |  | | **X** | **PRISMA FLOW DIAGRAM shows that the no. of records after duplication is 83. However, in the next stage, the record screened (52) and records excluded (32) indicates a total of 84 records, which is not matching with the earlier stage (n=83).**  **Details of components used for quality assessment of the included studies are not described.**  **The authors should mention the value of I-square (usually >25%) for deciding the heterogeneity level.**  **Figure 3 is a funnel plot, and it is not a forest plot as mentioned.**  **Further, in the funnel plot, it is clearly shown that all the prevalence values are outside the 95% confidence limits, indicating a clear publication bias. However, the authors stated that there is no publication bias, which is not true.**  **Since there is a publication bias, the p-value of the egger test should be significant (p<0.05). Therefore, the authors should carefully interpret the results.**  **Sample size & author (Figure 5) is not required.**  **Figures 7 & 8 are shown with eight studies; the reason for the inclusion of one more study is unclear.**  **In the Page 13, the statement, “**The possibility of women’s discontinuation of implanon for those women lacking counselling during service delivery were 2.54 times (OR: 2.34, 95%CI: 2.98, 2.77) more likely compared with women getting adequate counselling” is wrong due to wrong presentation of OR**.**  In the Page 13, the last sentence is stated as, “The possibility of discontinuation of implanon among women who lack the satisfaction of provided service was 4.42 times (OR: 4.42, 96% CI: 2.73, 7.15) more likely than their counterparts”. Why did the authors suddenly adopt 96% CI for this OR?  Meta-regression is a part of meta-analysis to determine the significant factors contributing to heterogeneity. Even though there is a high heterogeneity in the study, the authors did not carry out meta-regression to assess significant factors contributing to heterogeneity.  Limitation of study needs to be mentioned  **PRISMA checklist 2020 is missing** | | | |
| Are the conclusions and interpretations sound? | | **X** | |  |  | | | |
| Are the references properly cited? | |  | |  |  | | | |
| Is this a new/original contribution? | |  | | **X** | **It is a review work and cant be considered as a Research article.** | | | |
| Is it within the scope of the journal? | | **X** | |  |  | | | |
| Is the sample size adequate for the study? | |  | | **X** | **Only seven studies were included** | | | |

| **Overall the Paper is Rated:** | **(Excellent ---------7--------------------------------- Poor)**  10 9 8 7 6 5 4 3 2 1 |
| --- | --- |

**Sec. B: REFEREE'S RECOMMENDATIONS**	OTHER SPECIFIC CRITICISMS

| Accept with minor changes |  |  | Imperfect style |  |
| --- | --- | --- | --- | --- |
| Accept with major changes |  |  | Too long |  |
| Reject in current form, but may be resubmitted | X |  | References incorrectly presented |  |
| Reject, with no resubmission |  |  | Typographical and Grammatical errors |  |

YES

**PAPER TYPE**: **Research article	Review article**	Yes	**Letter article**

**Systematic review article	Case Report	Clinical Trial Study**

**Other**

**Have the following Reporting Guidelines been used by the authors in this study (if applicable)?**

- **Randomized Controlled Trials** – CONSORT – Consolidated Standards of Reporting Trials

| Yes |  |
| --- | --- |
|  |  |
|  |  |
|  |  |

- **Observational Studies** – STROBE – Strengthening the Reporting of Observational studies in Epidemiology
- **Systematic Review** **of Controlled Trials** – PRISMA – Preferred Reporting Items for

Systematic Reviews and Meta-Analyses

- **Study of Diagnostic accuracy/assessment scale** – STARD – Standards for the Reporting of

Diagnostic Accuracy Studies

- **Case Reports** – CARE – for case reports

**Does the manuscript comply for Ethical Guidelines (if applicable)?**

No

- Has the author provided the approval from local institutional review board? Or does the manuscript
  conform to the Helsinki Declaration on human experiments in the “Methods” section?
- Has the approval of institutional ethics committee been obtained by the author regarding

experimentation on animals or other species? Is the name of the species mentioned in the title

abstract and “Methods” Section of the article?

- In case of experimentation on human subjects, has informed consent been taken?

**Confidential Comments to the Editor (not for Transmission to Authors):**

The authors did not carry out adequate statistical analysis. It needs major revision and further review work

**Comments for the Authors *(continue on another sheet, if necessary)*:**

The comments given in the Evaluation sheet are applicable to authors also

**FIELD OF EXPERTISE OF REFEREE: Biostatistics**

**Name & Affiliation of Referee: Dr. Vanamail Perumal (Ex. Faculty), All India Institute of Medical Sciences, New Delhi, India**

P.Vanamail 16/02/2022

**SIGNATURE OF REFEREE / DATE**
