# Supplementary material for: Emergence of power and complexity in obstetric teamwork
Source: PLoS One. 2022 Jun 9;17(6):e0269711. doi: 10.1371/journal.pone.0269711 (PMC9182228; doi:10.1371/journal.pone.0269711)
Supplement: Attachment — Submitted filename: Reviewer Response.docx [file pone.0269711.s005.docx]

**Reviewer #1:**

**Dear Researchers, I was interested to read this from a methods point of view. I am not an expert in this area and provided a detailed review on methods leaving other sections to other reviewers. I felt currently that I wasn’t clear on the methods and some improvement was needed. I have identified this below. I wish you the best going forward.**

**Abstract
Methods
Please identify the paradigmatic stance and methodology selected.**

As described on P5L118, we used applied thematic analysis (ATA), as our study was mostly exploratory in nature. The ATA approach is “[…] a rigorous, yet inductive, set of procedures designed to identify and examine themes from textual data in a way that is transparent and credible” (Guest, MacQueen, & Namey, 2012). Drawing from a multitude of theoretical and methodological perspectives, its “[…] primary concern is with presenting the stories and experiences voiced by study participants as accurately and comprehensively as possible” (Guest et al., 2012). From a paradigmatic standpoint, our analytic process is leaning toward a positivist perspective, as we used various data reduction techniques. However, the act of identifying themes within text, among other components of the data analysis process, is itself a highly interpretive endeavor. Also, ATA embraces key elements of the interpretive school of thought, as it is continuously emphasized to always refer back to the raw data and caution against relying only on summarized forms (Guest et al., 2012).

**Please identify inclusion criteria.**

To be included in the study, participants had to be aged 18 years or older and belong to one of the following professions: Board-certified obstetrician or anesthesiologist, certified or registered nurse or midwife. We have expanded the manuscript (P6L138) to include this information that was so far only available in the appendix.

**Please consider methods used to obtain data, analysis approach undertaken.**

Since data collection for this manuscript was performed as part of a larger international project in Germany and Uganda, other data collection methods than individual interviews were discussed but then discarded due to logistical considerations. The analytic approach is delineated above.

**Methods main text**

**Appendix B seems to have more information, one concern for me was the volume of expected information here within the text e.g., there is no sampling technique named here, no consideration to sample size and why on the numbers, no consideration to a section on rigour or quality – you may mention this in appendix B but it is no good there.**

Thank you for this important remark. We have expanded the manuscript to give important information on the issues you raised for the reader without having to consult the appendix. P6L138 now reads: *Participants were recruited using convenience sampling as a type of non-probabilistic sampling (Elliot, Fairweather, Olsen, & Pampaka, 2016). Following a three-step procedure, first the target population was determined by the inclusion criteria specifying that participants had to be aged 18 years or older and belong to one of the following professions: Board-certified obstetrician or anesthesiologist, certified or registered nurse or midwife. In a second step, the sample frame was defined by the study site that this project was designed around. Third, individual enrolment was based on subject availability and accessibility, with a target sample size of 10-15 participants. The sample size was in part based on an estimated degree of data saturation, but also based on pragmatic considerations regarding the researchers’ time for as well as the scope of this project. Participants engaged in a semi-structured interview with one of the primary investigators. The interviews were conducted face-to-face, nobody except the participant and the researcher was present during the interviews.*

We also included a section on validity & reliability that reads (P8L164): *In addition to the aforementioned audit trail, we took the following steps to increase the validity of our research during various stages of the project:*

*The study design, including the format and questions of the semi-structured interview, was developed by both principal researchers together. This ensured familiarity with the research objectives and the envisioned methods for data acquisition.*

*The interviews were conducted by the same researchers that designed the study, therefore the purpose of each question was known as prerequisite for further inductive probing. During the interview phase, we constantly monitored and compared data as it was collected, so probing techniques could be aligned and overall data consistency enhanced.*

*Transcriptions were made to provide verbatim accounts of the data collection event, including the captioning of reactions (thought phases, pauses, laughs, sighs, etc.). In addition to providing more precise accounts of the interviews than mere field notes, we were able to capture and document rich, powerful and sometimes very emotional quotes that convey much more meaning than any analysis ever will.*

*A codebook was developed through multiple iterations. Also, coding was conducted together, so that ambiguities and reliability problems due to different interpretations could be immediately resolved.*

We were striving for a balance between required information and manuscript length and are therefore grateful for your help in calibrating the content.

**Line 101 – you call the work here a case study, but your title is an observational study? You also perform thematic analysis which in the results doesn’t seem to pull out information as case studies may? Consider clarifying this for the reader**

Thank you for this remark, we of course performed a case study and have corrected the title.

**Line 101 – you use reference 17 to name your approach a qualitative case study – however looking at this reference I could only find limited references to case study approaches. Can you identify unique characteristics of this methodology or give a better reference because a reader wont be able to understand the methodological process you are required to consider? Please remember you need to link your paradigm with methodology and quality criteria e.g., an interpretivists criteria could vary hugely compared to someone who assumes a critical realist criteria.**

We have changed the reference to a different book by the same author with extensive overview of the methodology. We also removed the “qualitative”, to clearly position the methodology as a case study, as there is an ongoing discussion around whether case study research is separate from qualitative research or not that we choose not to engage in. Our highlighting the fact that our research was primarily qualitative in nature is due to the medical tradition of prioritizing quantitative over qualitative research, and should be interpreted as our effort of improving acceptance of these methods that are widely and successfully applied in disciplines other than medicine. We are convinced that many a rich narrative is being lost in the all-too-common process of quantifying data for the sake of r-values and perceived objectivity in the medical field.

**I note you mention validity and reliability in your Appendix B this may be ok in some paradigms views but consideration is needed.**

See above

**Line 101 – do you consider a checklist like CARE or equivalent to aid the information here?**
We uses the COREQ checklist from the EQUATOR network, whose results are included in Appendix B

**Line 102 – what do you mean by a pre-approved interview schedule? How did you decide on the questions? Does it link to literature? Did you pilot this first and if so what changes were made? Was there any PPI involved?**

Pre-approved refers to the approval by the ethics board of the Heidelberg University Faculty of Medicine. The questions were decided by the principal investigators in an iterative process based on their own literature research and process of formulating the research question. They were not piloted first.

**You refer to Appendix A and within it cite Mansers work that splits the concept of TEAM work up – how does Table 1 in the text link to Table 1 in the appendix?**

They are not directly linked. As we explain in the appendix, the framework by Manser is one of the most cited frameworks to organize and understand teamwork in the dynamic domain of healthcare, and subsequently influenced our study design, research question and initial analysis. We did not want to explicitly ask about teamwork because the term might be biased for the participants. Instead, we prefered to ask about how participants perceive ‘good work’ or successful work. However, the work by Manser provided the framework for further probing.

**Analysis section follows – however for me the 8 stages of analysis in Appendix B can be named here and Appendix B needs to illustrate the application and examples of each stage as applied.**

We have expanded the Methods section to include a more detailed description of the analysis. It now includes (P6L132):

*The iterative process of data analysis consisted of the following phases:*

1. *Establishing clear analytic objectives: The main purpose of our study was to harness the narratives of practitioners and develop an understanding for their conceptualization of teamwork. This is highly exploratory in nature.*
2. *Data quality control and enhancement during data collection: During the interview process, the two researchers frequently exchanged experiences and compared notes regarding the interviews to control the quality of data gathered and ensure reliability, and to already familiarize oneself with the data and develop a ‘feel’ for recurring themes and ideas voiced by the participants.*
3. *Text segmentation and quality control: After transcription, interview data was segmented by questions as specified in the interview guide. This was later used to assess and compare the consistency of the questions asked and to provide a foundation for comparability. This segmentation was purely structural in nature and meant to support our methodological approach. We were especially conscious of the ongoing controversy regarding text segmentation and its potential for distorting context and meaning (Gibson & Brown, 2009), therefore the original interview dialogue was preserved and used for all coding and analysis of content.*
4. *Development of initial themes and codes: In the initial mapping of our data, both researchers together looked for representations of teamwork aspects as described by Manser (2009, see Table 2) in the participant’s narratives, while at the same time generating emergent themes and defining vague boundaries around them.*
5. *Development of a codebook and content coding: In the next iteration, themes were more closely defined, restructured, and a codebook developed for definite coding of the text (see S3 Appendix 3). Subsequently, all interviews were coded for content by both researchers together. This process was repeated twice, where the codebook was further refined, and analysis notes, ideas and memos were recorded for later analysis. It is important to note at this point that steps 3 – 5, while easily broken down into a logical sequence of events, can hardly represent distinct processes. Guest et al. (2012) note that “[…] the act of identifying a meaningful segment of text calls for some minimal representation of that meaning as a code, a note, a query, or a tag”. Subsequently, coding ideas come to mind even during the initial reading and structural segmentation of the text. On the other hand, codes that were conceived and hardly ever applied were later changed, or redefined, as the process (r)evolved.*
6. *First-order data analysis and comparison: Subsequently, we analyzed data to provide a description of how teamwork was constructed by our participants. Also, quantification methods were used, mostly to visualize code frequencies and aid the researchers in pattern recognition.*
7. *Second-order analysis: During the second-order analysis, the sometimes incomplete, sketchy or contradictory data is connected with theoretical literature with the aim of constructing theoretical explanations. According to Shkedi (2004), the purpose of these explanations is not necessarily the construction of “grand theory”, but rather a conversion and organization of the content and the connection with the researcher, as it is impossible to separate the inquirer from the inquired from a constructivist epistemological perspective. For us, this not only meant making sense of the analysis, but aligning the results with our own experience and understanding of the delivery of peripartum care.*
8. *Writing the report: Rather than a mere form of representation, writing “[…] plays an active part in the process of organizing, working with and analyzing data” (Gibson & Brown, 2009). It is also a way of crystallizing vague conceptions and connecting loosely formulated ideas, and presenting them to the reader through sound reasoning and exemplification.*

**Results
Clarity over type of methodology used is needed. Consideration to minor themes within major themes could be considered.**

We hope that our expansion of the Methods section has brought more clarity in regard to our methodology. (see above)

**Reviewer #3: When I was invited to review this manuscript it was especially the phrase “… we have yet to understand how the perception of teamwork … is shaped in a complex, multi-disciplinary environment” that raised my interest, as I thought I could relate to my own prior work on systems science around issues of dynamic complexity in the delivery of health care. But let me make a disclaimer: I’m not an obstetrician (not a clinician even) and neither do I have a background in andragogy or socio-anthropology. My comments are as follows.**

**Since this was an exploratory study I find it strange to see the Introduction section being concluded with the sentence “Based on our analysis we generate an understanding of teamwork as a phenomenon emerging from interpersonal relationships, complex relations of power, and the enactment of current quality management practices.” This begs the question whether the researchers really had an open mind in this study – or in other words: I’m a little suspicious that this study was conducted in this particular university hospital for a certain reason, possibly because of an issue (conflict?) that had come to the surface earlier – and if so, the authors might have had some experiences (preconceived ideas?) that guided this study in a particular direction, i.e. that of power imbalances among the members of the obstetrical team. I am not against an N=1 study, but I do feel that an explanation is required why the study was conducted at this particular hospital.**

Thank you for this important remark. To put things in a broader perspective, the data for this manuscript was obtained as part of a larger study that compared obstetric teamwork in Germany, as representative for an industrial nation with high socioeconomic standard, and Uganda, a developing nation and one of the poorest countries in the world. According to the World Health Organization (WHO), in 2015 the maternal mortality ratio in Uganda was 343 per 100.000 live births (0.34%), compared to 6 per 100.000 (0.06‰) in Germany, a factor of over 50. The neonatal mortality ratio was 19 per 1.000 live births (1.9%), compared to 2.3 (0.23%) in Germany (World Health Organisation, 2019). By examining teams that manage peripartum emergencies in different settings and cultures, the project aimed to explore how successful teamwork is constructed and perceived by those directly involved in patient care, and contrast these findings with traditional normative approaches. The study locations were chosen because both principal investigators (C. Neuhaus, D. Lutnaes) had respective ties to the institutions involved: Dr. Neuhaus as anesthesiologist at the University Hospital Heidelberg, Mr. Lutnaes as simulation instructor teaching fetomaternal care in Mbarara, Uganda.

Undeniably, personal experiences at both study locations led both PIs to formulate the research question and generate interest in the topic of how teamwork is enacted and perceived in the first place. As we elaborate in the methods and limitations section, however, we have tried to harness this potential source of bias in a transparent way. The conclusions drawn in the study, especially regarding power as an object of the discourse, are the result of this process, and have led us to be able to articulate dimensions of teamwork that we were unable to grasp, or describe, before.

We changed the conclusion of the introduction to better reflect the research question and not anticipate the results of our work.

**I am missing a study objective!**

See above; we tried to clarify this by changing the introduction to explicitly state the study objective and research question.

**The methods section claims that “the theoretical foundation of the study was that obstetric teamwork is the result of a balancing act in which multiple goal conflicts are continuously negotiated and managed right at the boundary of acceptable performance in a complex adaptive system”. While I agree that (obstetric) team work has multiple features, many of which may mutually influence each other, I’m not sure whether the term ‘multi-disciplinary’ applies. More importantly, however, a conceptual framework around teamwork dynamics from sectors other than obstetrics – or even from sectors outside healthcare – might have informed the study design. It appears that the five interview questions (which seem pretty general) and the fairly short duration of the interviews (30 minutes on average) did not allow for much depth.**

The attribute ‘multi-disciplinary’ was chosen because obstetric, peripartum teamwork involves several different disciplines of professional specialization, e.g. obstetricians, anesthesiologists, nurses and midwifes. Some argue that the term ‘inter-professional’ also applies to further highlight the collaboration of physicians and nurses; however we decided to focus on the different disciplines and explore their respective outlook. One explanation for the relative brevity of the interviews was that, as both the interviewer and the participants were highly skilled professionals within the domain, many explanations and introductory preliminaries could be skipped.

**The sentence “We explored this theory by gathering lived experiences of successful management of peripartum emergencies” further triggers me: why not investigate/evaluate emergency cases that were handled unsuccessfully (or less successfully) – which I believe are sometimes referred to as ‘adverse events’? Were there any adverse events at all in this particular hospital? If yes, could one not learn more from a less successfully managed emergency case (even if only one) than from cases that were managed well?**

Thank you for raising an important point. We explicitly refrained from focusing on adverse events, but rather decided to ask open questions and harness the participants’ perspectives on “work-as-is” or “work-as-done” (Shorrock & Williams, 2016). As apparent in our interview transcripts and most likely prompted by our fairly general, or neutral, questions, participants were free to choose the types of events they related to explain or underscore their respective points. This resulted in the mentioning of a wide variety of cases, a fact that we embraced in our effort of exploring obstetric work in a naturalistic approach. A variety of frameworks describe the potential benefit of this approach in an effort to learn not only from events that resulted in unfavorable outcomes, but from the (generally much more numerous) experiences where clinical work goes as planned – a philosophy more commonly known as “Safety I – Safety II” (Erik Hollnagel, 2014) that is a broadly accpted approach in resiliency circles (Erik Hollnagel, Braithwaite, & Wears, 2013; Woods, 2003) and among proponents of a Joint Cognitive Systems approach to understanding complex adaptive work environments (E. Hollnagel & Woods, 2005; Woods, Dekker, Cook, Johannesen, & Sarter, 2010). It is important to note that the distinction between ‘successful’ and ‘unsuccessful’ remains somewhat academic and is nowhere near dichotomous when viewed through a clinical and/or safety lens. In the domain of healthcare, boundaries between success and failure are subject of considerable debate, and causality is often unclear. The difference between process and outcome as object of analysis comes to mind, yet in a more consequent understanding, in a complex adaptive system, a perfectly safe process can have an adverse outcome, and a totally unsafe process can have a desired one. If we consider adverse events “unexpected deviations from an expected outcome” and focus our definitions, and way of thinking, on these events, we are increasingly dealing with things that are – for the most part – not happening (or in Hollnagel’s words “dynamic non-events” (2014)). Looking at safety as an ongoing, dynamic system property requiring continuous adaptation, however, refocuses our attention on processes in the workplace that include many more successful adaptations and robust ways to get work done instead of just the few incidents where something failed. Furthermore, it allows us to capture influences of history and culture that have been described in various theoretical frameworks, e.g. the constant “practical drift” (Snook, 2000) or “production of culture” (Vaughan, 2016) that happens within an organization while adapting to the necessities of everyday work.

**Methods: the topic of this study would lend itself perfectly for data collection methods other than, or at least complementary to individual interviews – for instance focus group discussion; or group model building (led by 2-3 moderators) with participants jointly developing a causal loop diagram. Has this been considered at all? I would have expected the authors to say something in the Study limitations about the method used in their study and possible alternative methods. The Study limitations subsection mentions “the use of additional data sources and triangulation” but it is not quite clear what this refers to.**

Since data collection for this manuscript was performed as part of a larger international project in Germany and Uganda, other data collection methods than individual interviews were discussed but then discarded due to logistical considerations. We have expanded the Limitations section to increase transparency in this regard. Triangulation refers to the technique to increase credibility of research, more specifically data collection triangulation and researcher triangulation (Guba & Lincoln, 1985). We were able to use the latter during the different phases of our analysis (Steps 2-7, see Appendix 2)

**In the results section: “… teamwork also becomes the framework for negotiation of many conflicts that originated elsewhere.” This statement cries for more detail: what kind of conflicts did the researchers come across, how did they present themselves, how did they affect clinical management, did they (negatively) affect the outcome of the performed obstetric procedures?**

The statement refers to (amongst others) the respective hierarchies or goal conflicts within the involved professions, the aspect of power and the aspect of quality vs. safety that is elaborated in the following paragraphs. We have expanded the manuscript to better connect these parts.

**The Discussion section seems to be a little detached from the Findings. As I said, I’m not an expert in andragogy or related disciplines, but I do think that the discussion on power imbalances could have reflected a bit more on factors such as expertise, formal hierarchy, claims to moral authority, and epistemic versus normative power.**

Thank you for this comment. We have expanded the discussion section to include more reflections on hierarchy and epistemic versus normative power (P16L400ff, P19L468ff, P20L495ff). This is a very fitting addition to Luke’s framework on power that we used in the discussion and reinforces the subtle interpersonal and interprofessional aspects we are trying to convey.

Elliot, M., Fairweather, I., Olsen, W., & Pampaka, M. (2016). non-probability sampling. Retrieved from <http://www.oxfordreference.com/view/10.1093/acref/9780191816826.001.0001/acref-9780191816826-e-0280>

Gibson, W. J., & Brown, A. (2009). *Working with Qualitative Data*. doi:10.4135/9780857029041

Guba, E. G., & Lincoln, Y. S. (1985). Naturalistic inquiry. In. Newbury Park, CA: Sage.

Guest, G., MacQueen, K., & Namey, E. (2012). *Applied Thematic Analysis*. doi:<https://doi.org/10.4135/9781483384436>

Hollnagel, E. (2014). *Safety-I and safety-II : the past and future of safety management*. Farnham: Ashgate.

Hollnagel, E., Braithwaite, J., & Wears, R. L. (2013). *Resilient health care*. Boca Raton, FL: CRC Press.

Hollnagel, E., & Woods, D. D. (2005). *Joint cognitive systems : foundations of cognitive systems engineering*. Boca Raton, FL: Taylor & Francis.

Manser, T. (2009). Teamwork and patient safety in dynamic domains of healthcare: a review of the literature. *Acta Anaesthesiologica Scandinavica, 53*(2), 143-151. doi:<https://doi.org/10.1111/j.1399-6576.2008.01717.x>

Shkedi, A. (2004). Second‐order theoretical analysis: a method for constructing theoretical explanation. *International Journal of Qualitative Studies in Education, 17*(5), 627-646. doi:10.1080/0951839042000253630

Shorrock, S., & Williams, C. (2016). *Human Factors and Ergonomics in Practice: Improving System Performance and Human Well-Being in the Real World*: CRC Press.

Snook, S. A. (2000). *Friendly fire : the accidental shootdown of U.S. Black Hawks over Northern Iraq*. Princeton, N.J.: Princeton University Press.

Vaughan, D. (2016). *The Challenger launch decision : risky technology, culture, and deviance at NASA*. Chicago ; London: The University of Chicago Press.

Woods, D. D. (2003). Creating foresight: How resilience engineering can transform NASA’s approach to risky decision making. *Work, 4*(2), 137-144.

Woods, D. D., Dekker, S., Cook, R., Johannesen, L., & Sarter, N. (2010). *Behind human error* (2nd ed.). Farnham: Ashgate.

World Health Organisation. (2019). Global Health Observatory (GHO) data. Retrieved from <https://www.who.int/gho/maternal_health/countries/en/>
